# Impact of reduced mycophenolate exposure on chronic lung allograft dysfunction incidence after lung transplant

**DOI:** 10.1016/j.jhlto.2024.100156

**Published:** 2024-09-06

**Authors:** Kaitlyn Grieves, Brian C. Keller, Georgina Waldman, Jacqueline E. Clark

**Affiliations:** a Department of Pharmacy, Massachusetts General Hospital (MGH), Boston, Massachusetts; b Division of Pulmonary and Critical Care Medicine, Massachusetts General Hospital (MGH), Boston, Massachusetts

**Keywords:** lung transplant, mycophenolate, rejection, CLAD, immunosuppression

## Abstract

**Background:**

Mycophenolate mofetil (MMF) is a key immunosuppression agent for lung transplant recipients (LTR); however, the side effects often lead to dose modifications. Kidney transplant literature has shown reductions in MMF dosing led to an increased incidence of rejection, but data are limited in LTR. The objective was to evaluate the impact of reduced MMF exposure on chronic lung allograft dysfunction (CLAD) in LTR within 36 months of transplant (TXP).

**Methods:**

This single-center, retrospective cohort analyzed LTRs who had an MMF dose reduction or hold ≥7 days between April 1, 2016 and October 31, 2019. LTR who died ≤1 month from TXP were excluded. The primary outcome was incidence of CLAD 36 months from TXP compared to the International Society for Heart and Lung Transplantation (ISHLT) registry data. Secondary outcomes were incidence of treated acute cellular rejection and characterization of MMF dose modifications.

**Results:**

Of 109 patients evaluated, 102 (93.6%) patients had 194 MMF dose modifications within 36 months of TXP, largely due to hematologic toxicities (74.7%). Before modification, 142 (73.2%) were receiving MMF 1,000 mg/day and 52 (26.8%) were receiving 500 mg/day. Incidence of CLAD was 36.4% at 36 months compared to 32.6% reported by ISHLT (*p* = 0.5216). Incidence of patients with decline in forced expiratory volume in 1 sec ≥10% was 45.1% at 36 months.

**Conclusions:**

In our cohort, most LTRs had an MMF dose modification within 36 months, yet CLAD incidence was consistent with rates reported in the ISHLT Thoracic Organ Transplant Registry. In contrast, more patients demonstrated reduced allograft function compared to post-TXP peak, consistent with “potential” CLAD.

## Background

According to the Organ Procurement and Transplantation Network, the number of lung transplants performed in the United States has continued to rise with 3,026 lung transplants in 2023. Improvements in immunosuppression management have positively impacted post lung transplant survival.^1^, ^2^

Despite immunosuppression advancements, there remains significant morbidity and mortality in this population. Chronic lung allograft dysfunction (CLAD) is the leading cause of death beyond the first year of transplant.^3^ CLAD is an umbrella term that describes substantial deterioration in lung function due to pathologic progression in the airway and parenchyma of the lung allograft. The International Society for Heart and Lung Transplantation (ISHLT) guidelines recommend that CLAD should be investigated when the forced expiratory volume in 1 sec (FEV_1_) declines by ≥10% from baseline (“potential” CLAD), and the diagnosis of “definite” CLAD is confirmed when FEV_1_ declines by ≥20% for at least 3 months. Risk factors for CLAD include reduced immunosuppression, acute and antibody-mediated rejection (AMR), and infection.^4^

The mainstay immunosuppression regimen for lung transplant recipients consists of tacrolimus, mycophenolate mofetil (MMF), and corticosteroids.^5^ MMF is an antimetabolite that inhibits T and B cell expansion by inhibiting purine synthesis.^6^ Due to its mechanism of action, MMF has significant adverse effects such as gastrointestinal intolerance, cytopenias, and infection. These adverse events can be severe enough to lead to preemptive dose reductions or discontinuation of MMF.

The kidney transplant literature has demonstrated that significant reductions in MMF dosing can lead to an increased incidence of rejection. One study by Vanhove et al showed that 47.9% of patients had a dose reduction or discontinuation with the primary reason being hematologic toxicities. Additionally, those whose doses were reduced ≥50% from baseline had a statistically significant increased risk of acute rejection but no effect on graft survival.^7^ Moreover, a systematic review by Su et al showed higher incidences of rejection and graft loss when MMF was reduced ≥30 days post-transplant vs <30 days post-transplant.^8^ Data showing the effects of MMF dose reduction or discontinuation in lung transplant recipients are lacking.

Here, we evaluate the impact of reduced MMF exposure on CLAD, treated acute rejection, and mortality in lung transplant recipients within 36 months of transplant.

## Methods

This was a retrospective cohort analysis conducted at a single academic medical center that included patients who underwent bilateral or single lung transplantation from April 1, 2016 to October 31, 2019. The inclusion criteria were all lung transplant recipients ≥18 years of age who were started on MMF 1,000 mg/day per the standardized protocol. The standard mycophenolate dosing during the study period was 1,000 mg/day due to historical challenges with infection and leukopenia. Patients who received a multiorgan transplant were transplanted at another center or died within 1 month of transplant were excluded. Dose modification was defined as a reduction or hold lasting ≥7 days. Patients were followed from the time of transplant to 36 months post-transplant. All data were extracted from the electronic medical record and collected using REDCap.^9^

### End-points

The primary outcome was incidence of CLAD 36 months after transplant. “Definite” CLAD was defined as a persistent decline in FEV_1_ ≥20% for more than 3 months and “possible” CLAD as a ≥20% decline for at least 3 weeks but less than 3 months. Patients with “potential” CLAD were defined as those with a decline in FEV_1_ ≥10% but <20% for at least 3 weeks.^4^ Secondary outcomes included incidence of treated acute cellular rejection and all-cause mortality at 3 years after transplant. Treated acute cellular rejection was defined as requiring methylprednisolone 125 mg equivalence or greater per day for at least 3 days for treatment of rejection. CLAD, rejection, and mortality end-points were compared to the 2019 and 2021 ISHLT International Thoracic Organ Transplant Registry Data (ISHLT Registry).^10^, ^11^ ISHLT Registry data reports data as bronchiolitis obliterans syndrome (BOS); however, due to a change in terminology, the present study utilizes the term CLAD for an all-encompassing term of chronic rejection.^4^ Additionally, our study looked at the indication for MMF dose reductions, time from transplant to MMF dose reduction, duration of dose reduction, incidence of AMR, and time from dose reduction to CLAD and/or rejection. AMR was defined as de novo donor-specific antibodies. Last, the study assessed the incidence of leukopenia, thrombocytopenia, and granulocyte colony-stimulating factor use as well as estimated glomerular filtration rate.

### Immunosuppression regimens

Our center’s immunosuppression protocol in lung transplant recipients during the study timeframe utilized tacrolimus with a goal trough of 10 to 14 ng/ml for the first year, 8 to 10 ng/ml for the second year, and then 5 to 8 ng/ml thereafter. At the time of the study, patients were maintained on MMF 500 mg twice daily post-transplant and a protocolized steroid taper that ended in prednisone 20 mg daily for the first 6 months, then prednisone 15 mg daily for the next 6 months until 1 year post-transplant, and then prednisone 10 mg daily thereafter. All transplant patients received standardized opportunistic infection prophylaxis with lifelong sulfamethoxazole-trimethoprim, valganciclovir for 12 to 18 months post-transplant, and antifungal therapy for 6 months post-transplant. The timing of lung biopsies and FEV1 monitoring were determined by the clinician.

### Data analyses

Continuous variables were summarized using mean and standard deviation or median and range, and categorical data were summarized using counts and percentages. Categorical variables were analyzed with chi-square tests with Yates’ correction. A *p*-value of 0.05 or less was considered statistically significant.

## Results

### Baseline characteristics

**Figure 1:**
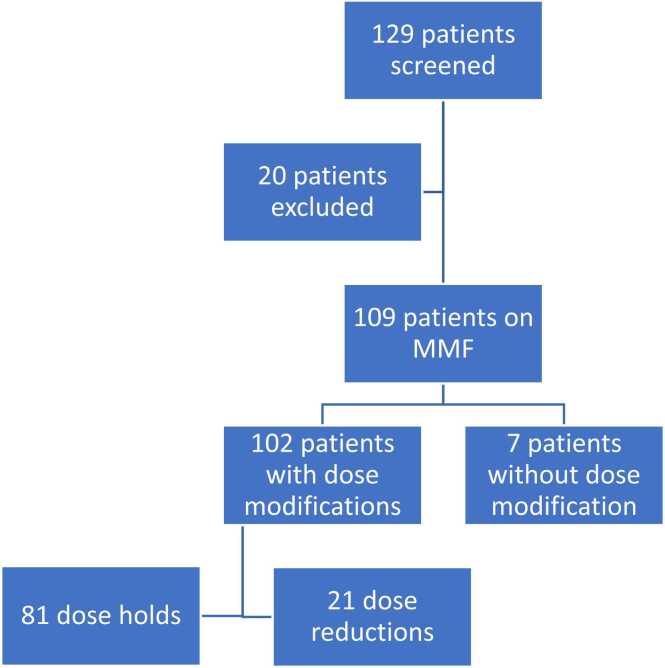
MMF dose modifications during study period. MMF, mycophenolate mofetil.

A total of 129 patients were screened for this study. Twenty patients were excluded: 9 (45%) did not receive the protocolized initial MMF dose of 1,000 mg/day, 5 (25%) were followed by another transplant program post-transplant, 3 (15%) received a multiorgan transplant, and 3 (15%) died within 1 month of transplant. One hundred and nine patients met the inclusion criteria for the final analysis (Figure 1). Baseline characteristics are presented in Table 1. Most patients (98.2%) received a bilateral lung transplant with 1.8% of patients receiving a retransplant. The most common indication for transplant was chronic obstructive pulmonary disease (26.6%) followed by idiopathic pulmonary fibrosis (23.9%).

**Table 1:** Baseline Characteristics

Characteristics	All patients initially on MMF (*n* = 109)
Age (years), mean ± SD	66 ± 18.1
Male, *n* (%)	68 (62.4)
Caucasian, *n* (%)	102 (93.6)
Body mass index (kg/m^2^), mean ± SD	25.1 ± 4.9
Bilateral lung transplant, *n* (%)	107 (98.2)
Initial transplant, *n* (%)	106 (97.2)
Indications for lung transplant, *n* (%)	
Chronic obstructive pulmonary disease	29 (26.6)
Idiopathic pulmonary fibrosis	26 (23.9)
Cystic fibrosis	19 (17.4)
Interstitial lung disease	19 (17.4)
Alpha-antitrypsin 1	5 (4.6)
Bronchiectasis	3 (2.8)
Nonspecific interstitial pneumonia	2 (1.8)
Sarcoidosis	2 (1.8)
Pleuroparenchymal fibroelastosis	1 (0.9)
Interstitial pneumonia with autoimmune features	1 (0.9)
Repeat transplant due to chronic lung allograft dysfunction	2 (1.8)
Induction agent, *n* (%)	
Basiliximab	23 (21.1)
No induction	86 (78.9)

Abbreviations: MMF, mycophenolate mofetil; SD, standard deviation.

### MMF dose modifications

Out of the 109 patients, 102 patients had a dose modification and 7 patients did not have a dose modification (Figure 1). There were 194 MMF dose modification events among the 102 patients within 36 months of transplant (64 patients had ≥2 dose modifications). The median time from transplant to first dose modification was 154 days (range 6-1,094), with 109 (56.2%) modifications occurring within 6 months of transplant. There were 142 MMF dose reductions in patients receiving MMF 1,000 mg/day. Additionally, there were 52 MMF discontinuations in patients with a previous dose reduction to 500 mg/day. Most dose modifications were made for hematologic toxicities (145 dose reductions, 74.7%), followed by infection (24 dose reductions, 12.4%), and gastrointestinal side effects (17 dose reductions, 8.8%). For dose modifications secondary to hematologic toxicities, the median time after dose modification to 2 subsequent white blood cell measurements ≥4 K/μl was 34 days (range 2-973 days). For dose modifications secondary to infection, 85.1% of dose reductions were done in the setting of viral infection and 14.8% were due to bacterial infections. Slightly more than half (51.9%) of viral infections were cytomegalovirus and 4 (14.8%) were respiratory syncytial virus. Table 2 shows a further breakdown of indications for dose modifications, pertinent labs at the time of modification, and concomitant medications.

**Table 2:** Mycophenolate Dose Modifications Within 36-Month Follow-Up

Characteristics	MMF dose modifications (*n* = 194)
Indications for dose modification, *n* (%)	
GI side effects only	15 (7.7)
GI side effects and leukopenia	1 (0.5)
GI side effects and thrombocytopenia	1 (0.5)
Leukopenia only	126 (64.9)
Leukopenia and infection	3 (1.5)
Leukopenia and neutropenia	9 (4.6)
Leukopenia and thrombocytopenia	2 (1)
Leukopenia and neoplasm	1 (0.5)
Thrombocytopenia only	4 (2.1)
Infection only	24 (12.4)
Neoplasm only	6 (3.1)
Pregnancy only	1 (0.5)
Other	1 (0.5)
Total number of dose modifications related to infection, *n* (%)	27 (13.9)
Viral infections, any, *n* (% of total infections)	23 (85.1)
Cytomegalovirus	14 (51.9)
Respiratory syncytial virus	4 (14.8)
Epstein-Barr virus	3 (11.1)
Adenovirus	1 (3.7)
BK virus	1 (3.7)
Bacterial infections, any, *n* (% of total infections)	4 (14.8)
Respiratory infection	1 (3.7)
Urinary tract infection	1 (3.7)
Sepsis	1 (3.7)
Tissue culture	1 (3.7)
WBC at time of MMF modification due to leukopenia (K/μl), mean ± SD	3.5 ± 0.7
ANC at time of MMF modification due to neutropenia (K/μl), mean ± SD	1.1 ± 0.7
Platelets at time of MMF modification due to thrombocytopenia (K/μl), mean ± SD	77 ± 34.8
Total number of dose modifications for hematologic indications, *n* (%)	142 (74)
G-CSF usage during dose modifications for hematologic indications, *n* (%)	39 (27.5)
Sulfamethoxazole-trimethoprim use at time of MMF modification, *n* (%)	99 (51)
Valganciclovir use at time of MMF modification, *n* (%)	116 (59.8)
Ganciclovir use at time of MMF modification, *n* (%)	0 (0)

Abbreviations: ANC, absolute neutrophil count; G-CSF, granulocyte colony-stimulating factor; GI, gastrointestinal; MMF, mycophenolate mofetil; SD, standard deviation; WBC, white blood count.

### MMF discontinuation and reduction subgroups

Out of the 194 dose modifications, 173 (89.2%) were dose discontinuations ≥7 days. In 59 of 173 cases of dose discontinuations, the patients did not resume MMF during the study period. After MMF discontinuation, in 15 of the 173 cases, the previous dose was resumed with a median time to resumption of 184 days (range 28-1,024 days). In 99 of the 173 cases, MMF was resumed at half-dose (500 mg/day) with a median time to resumption of 292 days (range 26-1,046 days). Only 53 of the 99 half-dose resumptions were increased back to 1,000 mg/day with a median time of 397 days (range 32-1,093 days) to reach the target dose.

**Figure 2:**
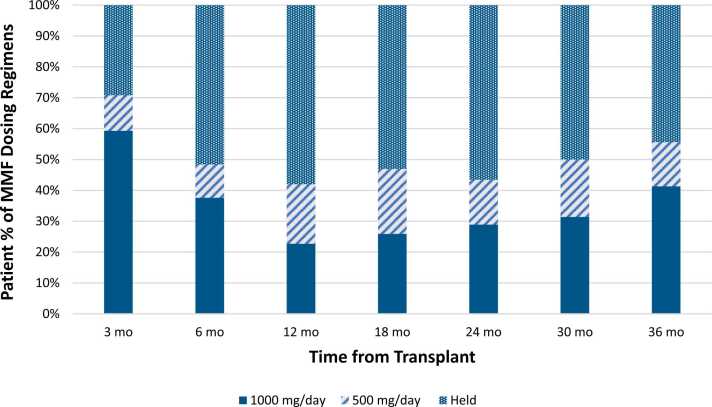
Mycophenolate mofetil dosing in the institutional cohort over the study period. MMF, mycophenolate mofetil.

In the dose reduction group, 21 (10.8%) of the 194 dose modifications were MMF dose reductions from 1,000 mg/day to 500 mg/day that lasted ≥7 days. Of these, MMF was directly resumed at 1,000 mg/day in 7 cases, while the other 14 patients remained on 500 mg/day. The median time to direct resumption of 1,000 mg/day was 790 days (range 63-1,123 days). At 12 months post-transplant, only 38 of 91 (41.8%) surviving patients were on MMF, with 22.7% of patients on 1,000 mg/day. Figure 2 shows the percentage of patients on different MMF dosing regimens over the study period.

### CLAD

None of the 7 patients without MMF dose modifications developed CLAD at 36 months. Out of the 102 patients with dose modifications, 88 patients had ≥2 FEV1 checked during the study period. Of 88 patients screened for CLAD, 32 (36.4%) patients developed CLAD at 36 months (24 with “definite” CLAD and 8 with “possible” CLAD). Twenty-one (23.8%) patients in our study had CLAD at 24 months (17 with “definite CLAD” and 4 with “possible CLAD”). Additionally, 9 (10.2%) patients had CLAD at 12 months (7 with “definite” CLAD and 2 with “possible” CLAD). There were 14 deaths (15.9%) related to CLAD within 36 months of transplant. When examining patients with “potential” CLAD, defined as a ≥10% but <20% decline in lung function from baseline, incidence of “potential” CLAD was 30.4% at 24 months and 45.1% at 36 months.

### Rejection

Fifty-three (52%) patients with MMF dose modifications experienced acute cellular rejection at any time within 36 months from transplant [median 2 episodes of rejection (range 1-8)] compared to only 2 (28.6%) patients without an MMF dose modification [median 2.5 (range 1-4)]. There were 115 treated acute cellular rejection episodes among the 102 patients with an MMF dose modification compared to 5 episodes among 7 patients who underwent no MMF dose modification. Only 23 (20%) were biopsy-proven rejection episodes in those with an MMF dose modification and 1 (20%) in those without an MMF dose modification, while the remainder received empiric treatment for suspected rejection without biopsy confirmation. Time between transplant and rejection was 449 days (range 13-1,096) in those with an MMF dose modification and 455 days (range 206-684) in those without an MMF dose modification. Out of 53 patients treated for acute cellular rejection, 31 (58.4%) patients had rejection within the first year. The average time from transplant to the first rejection episode within 12 months of transplant was 137 days (range 13-351). At 1 year, 8 (21.8%) patients on triple drug therapy (tacrolimus, MMF, and steroids) were treated for rejection. Additionally, 3 (100%) patients on tacrolimus, azathioprine, and steroids, 12 (26.1%) patients on tacrolimus and steroids, and 3 (100%) patients on a mammalian target of rapamycin inhibitor and steroids experienced rejection at 1 year in our study. Last, 12 (11.8%) patients experienced AMR within 36 months for those with an MMF dose modification, but there were no cases of AMR in patients without MMF dose modifications.

### Mortality

Thirty-four (33.3%) patients who underwent MMF dose modification died within 36 months of transplant, while none of the 7 patients without MMF dose modifications died during the study period. The cumulative incidence of death was 11 (10.8%) patients at 12 months and 24 (23.5%) patients at 24 months.

## Discussion

Our program utilizes a 3-drug immunosuppression regimen for lung transplant recipients consisting of tacrolimus, MMF, and steroids. Previous lung transplant literature shows that a majority of institutions utilize 2,000 to 3,000 mg of MMF per day; however, during the study period, our center used a lower dose of MMF consisting of only 1,000 mg/day.^12^, ^13^ In kidney transplants, the reported incidence of hematologic side effects with MMF ranges between 11% and 50% with increased incidences at higher doses.^14^ However, our study showed that even with low-dose MMF, most patients experienced an MMF dose reduction or hold for ≥7 days, mainly due to hematologic side effects.

**Figure 3:**
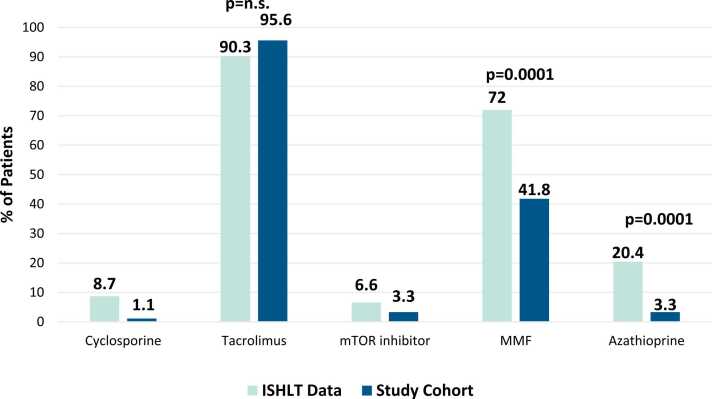
Percentage of patients on each immunosuppression agent at the time of 1-year post-transplant follow-up. ISHLT, International Society for Heart and Lung Transplantation.

Due to a low number of patients without MMF modifications, we were unable to compare outcomes for transplant recipients with MMF modifications compared to no MMF modifications within our own institution. Thus, we utilized the 2019 and 2021 ISHLT Registry data as a comparator group.^10^, ^11^ When compared with the ISHLT Registry, only 38 of 91 (41.8%) surviving patients in our cohort were on MMF at 12 months post-transplant compared to 13,800 (72%) patients in the ISHLT Registry (*p* = 0.0001, Figure 3).^10^ Additionally, no statistically significant differences were noted in the cumulative incidence of CLAD in this study compared to BOS in the ISHLT Registry within 36 months of transplant (36.4% vs 32.6%; *p* = 0.5216).^10^ Neither were differences in CLAD noted at 12 and 24 months post-transplant (10.2% vs 9.2%, this study vs the ISHLT Registry [*p* = 0.8781] and 23.8% vs 21.5% [*p* = 0.6775], respectively).^10^ Exactly 15.9% of patients in our study had a CLAD-related death within 36 months of transplant compared to 25.5% in the ISHLT Registry (*p* = 0.0535).^10^

For our secondary end-points, we found 31 (30.4%) patients had treated rejection within the first year, which is comparable to 21,030 of 79,060 patients (26.6%) with treated rejection reported by the ISHLT Registry (*p* = 0.4475).^11^ When comparing immunosuppression regimens of patients with treated rejection vs no rejection, 21.8% of patients were on tacrolimus, MMF, and steroids at 1 year in our cohort vs 23.8% of patients reported by the ISHLT Registry (*p* = 0.8323). Additionally, all 3 patients on tacrolimus, azathioprine, and steroids in our study experienced rejection at 1 year compared to 28.5% from the ISHLT Registry (*p* = 0.0356).^11^ Finally, there was no significant difference in cumulative mortality within 36 months of transplant in patients in our study compared to the ISHLT Registry (33.3% vs 29.7%, respectively, *p* = 0.4950).^11^

Most data for MMF use in lung transplant patients are extrapolated from transplant of other organs due to the small number of trials in the lung transplant population. One small study by McNeil et al showed an incidence of BOS at 3 years of 27% with MMF and 25% with azathioprine (*p* = 0.70). In this study, patients were maintained on MMF 3,000 mg/day for the first 3 months, then decreased to 2,000 mg/day.^15^ Based on the difference in MMF dosing, our patients should have been at higher risk for CLAD due to the frequency and duration of MMF dose holds or reductions. Further, when MMF was stopped in our patients, it was not replaced with an alternative immunosuppression agent such as azathioprine or mammalian target of rapamycin (mTOR) inhibitor. Despite these differences, our results did not show a difference in CLAD incidence at 36 months. Interestingly, our study showed the majority of our treated acute cellular rejection episodes were not biopsy proven. Empiric courses of high-dose corticosteroids may have augmented the overall net immunosuppression in our patients in whom MMF was held or reduced. Additionally, an increasing number of patients were able to tolerate MMF 1,000 mg/day starting 18 months post-transplant. These 2 factors may have helped to ameliorate the risk of CLAD.

The 2019 ISHLT Chronic Lung Allograft Dysfunction consensus report recommends that lung allograft dysfunction should be investigated when FEV1 declines ≥10% (“potential” CLAD).^16^ Our study shows that patients with a lower overall baseline MMF dose or those with a high number of MMF dose modifications may be at risk of “potential” CLAD development at 36 months. The higher incidence of patients with “potential” CLAD rather than confirmed CLAD may be due to the short 36-month follow-up period of our study. Only 40.7% of patients were on azithromycin and 64.4% of patients were on a statin for CLAD prevention at the time of MMF dose modification. These contributors may confound our reported CLAD incidence.^3^

Due to the retrospective nature of this study, this study was limited by documentation found in the electronic medical record as well as the inability to capture other risk factors for CLAD such as subtherapeutic tacrolimus levels and nonadherence to medications. Additionally, there may be a role for utilization of MMF therapeutic drug monitoring in patients on nontraditional dosing, though more data are needed to understand the efficacy correlation of MMF AUC levels in the lung transplant population.^17^ When using registry data as a comparator, there are additional limitations. Due to the high incidence of adverse effects with MMF, it is possible that MMF dose adjustments were included in the registry data. Additionally, there may be center-specific differences in how the incidence of CLAD is reported to the ISHLT Registry. Finally, the schedule of surveillance with pulmonary function testing and lung biopsies may have contributed to under reporting of rejection incidence in our cohort. A large, prospective randomized controlled trial with a longer follow-up period is necessary to evaluate the effects of lower MMF dosing more fully on CLAD development, and more data are needed to identify the optimal MMF dose to prevent CLAD.

## Conclusion

To our knowledge, this study evaluates the impact of reduced MMF dosing in lung transplant patients. In our cohort, most lung transplant recipients had an MMF dose modification within 36 months of transplant. The occurrence of CLAD in this retrospective cohort is in line with expected CLAD incidence per the ISHLT registry despite a high frequency of MMF dose holds or reductions; however, we found a higher incidence of patients meeting the criteria for “potential” CLAD. When MMF is held or reduced for a prolonged period of time, lung allograft function should be monitored closely to screen for CLAD development.

## Author contributions

Kaitlyn Grieves: conceptualization, methodology, formal analysis, investigation, writing—original draft, writing—review and editing, visualization. Brian C Keller: writing—review and editing, supervision. Georgina Waldman: conceptualization, methodology, validation, writing—review and editing, supervision, project administration. Jacqueline E. Clark: conceptualization, methodology, validation, writing—review and editing, supervision, project administration.

## Disclosure statement

The authors declare the following financial interests/personal relationships which may be considered as potential competing interests: Brian C. Keller reports a relationship with CareDx Inc. that includes consulting or advisory and funding grants. Brian C. Keller reports a relationship with Zambon Pharmaceutical Laboratories that includes consulting or advisory. Brian C. Keller reports a relationship with Natera, Inc. that includes consulting or advisory. Georgina Waldman was employed by Massachusetts General Hospital at the time of the research. Georgina Waldman reports a relationship with Takeda Pharmaceuticals USA Inc. that includes employment. The other authors declare that they have no known competing financial interests or personal relationships that could have appeared to influence the work reported in this paper.
